# Psychosocial characteristics are associated with adherence to dietary, drugs and physical activity recommendations amongst cardiovascular disease patients in Lebanon

**DOI:** 10.1371/journal.pone.0287844

**Published:** 2023-10-24

**Authors:** Melodie Al Daccache, Laila Al-Shaar, Abla Mehio Sibai, Hussain Ismaeel, Kamal Badr, Lara Nasreddine

**Affiliations:** 1 Faculty of Agricultural and Food Sciences, Department of Nutrition and Food Sciences, American University of Beirut, Beirut, Lebanon; 2 Faculty of Health Sciences, Department of Epidemiology and Population Health, American University of Beirut, Beirut, Lebanon; 3 Faculty of Medicine, Department of Public Health Sciences, Pennsylvania State University, State College, Pennsylvania, United States of America; 4 Department of Nutrition, Harvard T.H. Chan School of Public Health, Boston, Massachusetts, United States of America; 5 Vascular Medicine Program, American University of Beirut, Beirut, Lebanon; 6 Medical Services, Aman Hospital, Doha, Qatar; 7 Department of Internal Medicine, American University of Beirut Medical Center (AUBMC), Beirut, Lebanon; Shahrood University of Medical Sciences, ISLAMIC REPUBLIC OF IRAN

## Abstract

Cardiovascular diseases are increasing at an alarming rate worldwide, reaching epidemic proportions in countries of the Eastern Mediterranean Region, including Lebanon. Despite the growing number of patients suffering from cardiovascular diseases in Lebanon, there is scarce data on whether cardiac patients adhere to therapeutic dietary guidelines, drug prescriptions, and physical activity recommendations and whether such adherence differs according to sociodemographic, lifestyle, or psychosocial characteristics. A cross-sectional study was conducted among 367 Lebanese adult cardiovascular disease patients admitted for hospitalization at various hospital sites in Lebanon. Electronic medical records and a multi-component questionnaire were used to collect information on patients’ characteristics. Dietary assessment was performed using a culture-specific validated food frequency questionnaire, and physical activity levels were assessed using the international physical activity questionnaire (IPAQ). Mental well-being was assessed based on the validated five-item well-being index (WHO-5), and drug adherence was evaluated using the Morisky medication adherence scale (MMAS-8). The majority of the patients were males (67.8%), overweight or obese (74%), smokers (62.1%), and unemployed or retired (54.5%). Almost 35% of the patients were lonely, and nearly one fourth were at a high risk of poor mental health. Approximately 43%, 70%, and 52% of the patients were found to have poor adherence to diet, drug, and physical activity recommendations, respectively. A lower sense of mental well-being was a significant predictor of low dietary and drug adherence. Surprisingly, overweight and obesity were associated with higher odds of dietary adherence. Male gender was positively associated with physical activity while loneliness was inversely associated with physical activity. This study showed that adherence to diet, drug, and physical activity recommendations was low in this patient population and identified several non-clinical characteristics that may affect adherence. These findings highlighted the need for considering patients’ psychosocial characteristics in the treatment of patients with cardiovascular diseases.

## Introduction

The prevalence of non-communicable diseases (NCDs) is rapidly escalating worldwide, with a particular increase in low and middle-income countries [1]. More specifically, cardiovascular diseases (CVDs) are considered the leading cause of mortality, contributing to an estimated 31% of death globally [1,2]. In Lebanon, a small country in the Eastern Mediterranean Region (EMR), NCDs are estimated to account for 91% of all deaths, of which 47% are attributed to CVDs [2]. According to the World Health Organization (WHO), the age-adjusted death rate from coronary heart disease (CHD) reached 214.24 per 100,000 people in Lebanon, ranking the country in the 20^th^ place worldwide [3].

The etiology of CVDs involves a complex interplay between genetic predisposition and environmental factors [4] that may increase the risk of the disease. Lifestyle and behavioral risk factors are among the leading modifiable risk factors that can modulate the development and progression of CVDs [5]. In fact, the American Heart Association released a scientific statement in 2015, calling for greater attention to the social and behavioral determinants of CVD in different parts of the world [6].

Like other countries of the EMR, Lebanon is currently witnessing the nutrition transition with its characteristic shifts in diet, lifestyle, and body composition [7]. Amongst other environmental and behavioral risk factors, physical inactivity and the shift from the traditional diet to an energy-dense diet rich in sugar, fat, and animal-based products may explain the increasing burden of CVDs in Lebanon [7]. An energy-dense diet can induce excessive weight gain, leading to increases in blood glucose and triglyceride levels, coupled with decreases in HDL-cholesterol and increases in blood pressure and inflammatory markers [8]. Hence, in addition to pharmacological interventions, therapeutic dietary strategies represent an integral component of treating patients with CVDs [9]. Lifestyle strategies, including regular physical activity, decreasing sedentary time, and smoking abstinence, are also crucial for treating CVDs [10]. Despite ample evidence on the therapeutic effects of such strategies and interventions [10], there is a scarcity of evidence on whether patients with CVDs adhere to their therapeutic dietary guidelines, drug prescriptions, and physical activity recommendations and whether such adherence differs according to certain sociodemographic and lifestyle characteristics [11].

More recently, there has been an increasing interest in whether patients’ adherence to therapeutic strategies may be modulated by certain psychosocial characteristics. In fact, anxiety and depression are known to decrease the subject’s overall sense of mental well-being, thus potentially adversely impacting the patient’s quality of life and self-care behaviors [12]. It has therefore been suggested that anxiety and/or depression in CVD patients may be contributing to a substantial decrease in their adherence to drug prescriptions, and dietary as well as physical activity recommendations. This may therefore worsen the patients’ long-term prognosis and significantly increase their recovery period [13,14].

Acknowledging that CVDs are the leading cause of hospitalization amongst older adults in Lebanon [15], and considering the significant human and financial costs of hospitalization for these patients, there is a crucial need to better understand the spectrum of nonclinical characteristics of CVD patients and to identify the factors that may enhance adherence to dietary, drug and physical activity recommendations [11]. It is in this context that this study was conducted with the aims of 1) investigating the adherence of a sample of Lebanese CVD patients to dietary guidelines, prescribed drug regimens, and physical activity recommendations and 2) examining the association between adherence and psychosocial, socio-demographic as well as lifestyle characteristics.

## Materials and methods

This is a cross-sectional study of cardiac patients admitted for hospitalization in Lebanon. Data collection took place in four different hospitals in Lebanon as part of the parent study titled “Lebanese Cardiovascular /Cerebrovascular Health Cohort” (LC2HC) which was conducted between 2016 and 2018. The four hospital sites included the American University of Beirut Medical Center (AUBMC) (located in the capital Beirut; more than 400 bed-capacity) [16], Labib Medical Center (LMC) (located in the South of Lebanon; 137- bed capacity), AinWazein Hospital (AWH) (located in Mount Lebanon; 139 bed- capacity), and Centre Hospitalier du Nord (CHN) (located in the North of Lebanon; 110 bed- capacity). Although AUBMC is considered the largest academic tertiary care center in the country [16] and a major referral center for patients from various areas in Lebanon (44,728 inpatients annually) [17], we opted to enroll participants from 3 additional hospitals located in different governorates in Lebanon as a strategy to improve the diversity of the sample in terms of socioeconomic and cultural backgrounds. The World Health Organization (WHO) sample size calculator (N = Z^2^p (1-p)/d^2^)) was used to estimate the study’s sample size, assuming that Z = 1.96, p = 42% (prevalence of drug non-adherence as reported in a meta-analysis conducted among cardiac patients in 76 developing countries) [18] and d = 0.05. Accordingly the sample size was estimated at n = 375.

Convenience sampling was used in this study. Any Lebanese adult patient aged at least 18 years, conscious, with no previous diagnosis of mental illness, and admitted to cardiac catheterization and/or critical care unit (CCU) for cardiovascular reasons such as chest pain, high blood pressure, stroke, stenosis or angiography at AUBMC, AWH, LMC, or CHN hospitals during the study period was eligible and invited to participate in the study. Patients who were included in the study were mainly diagnosed with Acute Coronary Syndrome (ACS), Coronary Artery Disease (CAD), stable angina, stroke, high blood pressure, or stenosis. Patients aged 18 years or less, non-Lebanese or those who were unconscious were excluded.

Subjects were first informed about the study by their treating physician. Patients who expressed interest in participating in the study were then approached by a trained research assistant who obtained written informed consent and enrolled the participant in the study. A total of 400 subjects were enrolled in the study, from which a sample of 367 patients was included in the current analysis (n = 33 were excluded from the current analysis due to missing data). The approval to conduct the study was obtained from the Institutional Review Board (IRB) at the American University of Beirut (AUB), CHN, LMC, and AWH before the initiation of fieldwork. All participants provided written informed consent prior to their enrollment in the study.

Data collection was based on the administration of a multi-component questionnaire to patients admitted for hospitalization as well as the review of medical charts to collect anthropometric characteristics. The questionnaire inquired about demographic and socio-economic characteristics, psychosocial attributes, lifestyle characteristics, dietary intakes, and drug adherence. Within the hospital premises, the questionnaire was administered to patients through face-to-face interviews conducted by trained interviewers. For each subject, the interview took about 30–40 minutes to complete the questionnaire.

### A. Measures

#### 1 Demographic and socio-economic characteristics

The demographic characteristics included information regarding age, gender, marital status (married or not married), and site of recruitment (AUBMC, CHN, AWZ, and CHN). Socioeconomic characteristics included educational attainment (illiterate/elementary, intermediate or secondary/university), employment status (full-time, part-time, retired/unemployed), housing status (owned or rented), and perceived adequacy of household income (enough or not enough). The crowding index (C.I.) was calculated based on the total number of residents per household over the total number of rooms (excluding the bathrooms, garages, and open balconies). The crowding index is a proxy measure of socioeconomic status (SES), with an index > 1 indicating an overcrowded household [19].

#### 2 Anthropometric measurements

Anthropometric measurements were collected from patient’s medical charts and included height (cm), current body weight (kg), and waist circumference (WC). Body mass index (BMI) was classified as underweight <18 kg/m^2^, normal weight 18.5–24.9 kg/m^2^, overweight 25–29.9 kg/m^2^ or obese > = 30 kg/m^2^ [20]. Abdominal obesity was defined as waist circumference (WC) >94 cm in men and >80 cm in women as classified by the International Diabetes Federation cut-offs for Middle-Easterns [21].

#### 3 Psychosocial characteristics

Psychosocial attributes, including loneliness and mental well-being, were assessed. The evaluation of loneliness was based on the validated three-item loneliness scale that was initially developed by the University of California Los Angeles (UCLA) [22–24]. Participants were asked the following (1) “how often do you lack companionship?”, (2) “how much do you feel lonely?”, (3) “how much do you feel that there are people you can talk to?” and responses ranged from 1 = hardly ever or never, 2 = some of the time, and 3 = often. The scores for each patient were summed to give a possible score from 3 to 9 points [23]. The least lonely patients were identified based on a score ranging between 3 and 5 points, while lonely patients were identified based on a score ranging between 6 and 9 points. The scores of the third question were reversed, a higher score indicating greater loneliness.

Mental well-being was assessed using the World Health Organization five-item- well-being index (WHO-5) that was previously validated in screening for depression [25–27]. The WHO-5 item questionnaire was previously validated in Lebanon [28]. A cut-off point of less than 13 determines a decreased sense of well-being and a higher risk of depression [28]. Mental health state was measured by summing responses to the following questions: How much during the last month (1) “Have you been a happy person?”, (2) “Have you felt so down in the dumps that nothing could cheer you up?” (3) “Have you felt calm and peaceful?”, (4) “Have you been a very nervous person?”, (5) “You felt downhearted and blue?”. Each answer was scored on a scale from 0 to 5 points, with 0 coded for “all of the time”, 1 for “most of the time”, 2 for “good bit of the time”, 3 for “some of the time”, 4 for “a little of the time” and 5 for “none of the time”. The total score was calculated by reversing the answers to the first and third questions and summing the scores to a scale ranging from 0 to 25 points [28]. Higher scores indicate an increased sense of well-being.

#### 4 Lifestyle characteristics

The assessment of lifestyle characteristics focused on smoking history (current, former, or never smoked) and physical activity levels (low, moderate, or high).

Smoking history was assessed using a four-item questionnaire for cigarette and waterpipe smoking. The questions were related to (a) cigarette smoking status (current, former, or never), (b) frequency of cigarettes smoked per day (for current smokers only), (c) waterpipe smoking status (current, former, or never), and (d) frequency of waterpipe smoked per week (for current smokers). For the frequency of cigarette and waterpipe smoking, the options were categorized based on prior research [29,30]: the frequency of cigarette smoking per day was trichotomized into “1–14”, “15–24,” and “25+”. For waterpipe smoking per week, the options were categorized into “≤ 2 times”, “3–6 times”, or “daily”. Only patients who reported being current cigarette or waterpipe smokers answered the question related to the frequency of cigarette or waterpipe consumption.

Physical activity was assessed using the long version of the International Physical Activity Questionnaire (IPAQ) during the one-on-one interview. The IPAQ was previously validated for use among the Lebanese population [31]. The long IPAQ form consists of a 7-day recall questionnaire that covers four domains of PA, (1) Leisure time PA, (2) domestic and gardening (yard) activities, (3) work-related PA, and (4) transport-related PA. In each domain, the frequency and duration of time spent in each vigorous and moderate activity was recorded. The frequency and duration of walking time were included in the work, transportation, and leisure time domains. Activities that lasted at least 10 minutes were taken into account. Participants with missing data on the frequency or duration of any domain were considered non-active for that specific domain. The energy expenditure of an activity was expressed in metabolic equivalent tasks (METs) [32], and the following MET estimates were taken from the IPAQ protocol: 3.3 for walking, 3 for moderate domestic/inside chores activities, 4 for moderate PA, 5.5 for vigorous PA in the yard chores or garden, and 8 for vigorous PA [33]. The levels of physical activity were then classified into “low”, “moderate” and “high” according to the MET-min/week, duration of time, and frequency of PA [33].

#### 5 Dietary intake assessment

Dietary intake was assessed using a culture-specific semi-quantitative food frequency questionnaire (FFQ) that included 112 food items, and which was designed to estimate food intake over the past year. This FFQ has been recently validated for assessing nutrient intakes among adults in Lebanon [34]. The FFQ was administered during a one-on-one interview with patients admitted to AUBMC only, given that dietary assessment expertise was available solely in this hospital site.

For each food item or beverage included in the FFQ, the frequency of consumption as reported by the individual was converted to daily intake [35]. The Nutritionist Pro 1.2 software (Axxya Systems LLC, Stafford, TX, USA) was used to estimate the energy and nutrient content of the various food items included in the FFQ. For culture-specific/traditional food items, recipes were added to the Nutritionist Pro database based on a local cookbook [35]. Energy and nutrient content were calculated per gram for each food item on the FFQ list. Individual daily energy intake was then computed by summation of the respective products of the quantity consumed and the energy per gram value for each food item [36]. The same procedure was used to determine the daily nutrient intakes [37]. The percent contribution of the various macronutrients to energy intakes was also estimated.

The dietary intake of the study participants was evaluated based on the American Heart Association (AHA) guidelines, which specify the recommended intake for ten food groups and ten subgroups according to different energy intake levels [38]. Three food groups (solid fats, added sugars, and unsaturated oils) and one sub-group (beans & peas) were excluded from the current analyses due to data unavailability, yielding a total of 16 food groups and subgroups. The intakes of food groups (fruits, vegetables, grains, protein food, and dairy) in addition to whole grains and other grains were calculated in cups or oz equivalent per day. The intakes of food subgroups (dark green, red, starchy and other vegetables, lean meat, fish, and nuts) were calculated per cup or oz equivalent per week. The daily intakes of fiber and sodium were also determined in g/d.

Based on the AHA recommendations, all the food groups and subgroups were included in the scoring, except for the group “other grains” (biscuits, white bread, cereals, kaak, pizza, pasta, rice, etc.) which was later excluded given that it does not contribute to a healthy dietary patten [38]. As such, a total of 15 food groups, subgroups, and nutrients were included in the scoring system, and the intake was compared to the AHA recommended levels. Patients were assigned a score of 1 if the consumption was equal to or above the recommended value for each food group, subgroup, or nutrient according to their daily energy intake, and 0 if otherwise [38]. Only the score for sodium intake was reversed, and patients were given a score of 1 for an intake equal to or below the upper limit and a score of 0 for an intake above the upper limit. The scores of all the components were summed to give a score ranging from 0 to 15, with the higher score indicating higher adherence.

#### 6 Assessment of drug adherence

The assessment of drug adherence was based on the previously validated eight-items Morisky Medication Adherence Scale (MMAS-8) which inquires about drug habits retrospectively [39,40]. The tool consists of the following questions: (1) “do you sometimes forget to take your pill?”, (2) “Thinking over the past two weeks, were there any days when you did not take your medicine for reasons other than forgetting?”, (3) “have you ever cut back or stopped taking your medicine without telling your doctor because you felt worse when you took it?”, (4) “when you travel or leave home, do you sometimes forget to bring along your medicine?”, (5) “did you take all your medicine yesterday?”, (6) “when you feel like your symptoms are under control, do you sometimes stop taking your medication?”, (7) “taking medicine every day is a real inconvenience for some people. Do you feel hassled about sticking to your treatment plan?”, (8) “how often do you have difficulty remembering to take all your medicine?”.

The total score was calculated according to an already published method, in which all items were summed to give a score ranging from 0 to 8 [40]. The scores were dichotomized into two levels of drug adherence, high adherence for patients with a score of 8, otherwise non-adherent [41].

### B. Statistical analysis

Dietary intakes were analyzed using the Nutritionist Pro Software to determine daily energy and nutrient intakes. Median intakes of nutrients and food groups were computed after the exclusion of implausible total energy intake values (>6000 or <500 Kcal/day) [42] yielding a sample size of 129 subjects. Higher adherence to the AHA recommendations was defined as a score equal to or above 5 (≥ median) and lower adherence as a score below 5 (< median).

Based on the IPAQ, high PA was defined based on the following criteria: vigorous activity for at least three days achieving at least 1500 MET-min/week, or at least seven days of any combination of walking, moderate or vigorous activity achieving a minimum of 300 MET-min/week [33]. Moderate exercise was defined as at least three days of vigorous activity of at least 20 min/day, or at least five days of moderate activity and/or walking for at least 30 min/day, or at least five days of any combination of walking, moderate and vigorous activity achieving at least 600MET-min/week [33]. Patients who were not classified in the vigorous or moderate PA level were assigned to the “low” category [33]. PA was then dichotomized into two-category variables: low level versus moderate to high level.

Data analysis was conducted using the Statistical Package for Social Sciences (IBM SPSS, version 23 for Mac). Given the non-normal distribution of data, as evaluated using the Shapiro-Wilk test of normality, patients’ characteristics were presented as median and interquartile range (Q1, Q3) for continuous variables and frequencies and percentages for categorical ones. Bivariate analyses using chi-square test or Fisher exact tests were used for comparing categorical variables across the different subgroups defined by levels of adherence to diet, drug, and physical activity recommendations.

Multiple logistic regression analyses were performed with each of the drug adherence (low/moderate versus high drug adherence), dietary adherence (low versus high), and physical activity level (low versus high/moderate) as dependent variables, and sociodemographic, lifestyle, and psychosocial characteristics as independent variables. The models were adjusted for covariates that were identified from the literature on known associations with the dependent variables [43–46]. These covariates encompassed socio-demographic, anthropometric, behavioral and psychosocial characteristics [43–46]. Multicollinearity between the independent variables was checked, using the variance inflation factor (VIF), and a VIF <10 was considered to show no collinearity between the variables used in the model [47]. As such, the covariates that were adjusted for included age, marital status, income status, housing status, BMI, physical activity, smoking status, and loneliness level [43–46]. The odds ratio and the corresponding 95% confidence interval (CI) were used to evaluate the strength of these associations.

All reported p-values were two-sided and statistical significance was defined at the 5% level for all statistical tests. Missing data ≤ 5% were imputed to the median after stratifying by gender, age, and BMI.

## Results

### A. Sociodemographic, anthropometric, lifestyle, psychosocial, and behavioral characteristics of the study sample

#### 1 Sociodemographic, anthropometric, and lifestyle characteristics of the study sample

The total sample size included in this study was 367 participants, from which 193 participants (52.6%) were recruited from AUBMC and 150 (40.1%) from CHN hospital. Recruitment was suboptimal in AWH and LMC with only 24 patients from these two hospitals. Baseline socio-demographic, anthropometric, and lifestyle characteristics are shown in Table 1 for the total study sample. Of the study sample, 70.8% were males (*n* = 260), and 29.2% were females (*n* = 107), with a median age of 67 years (57, 74). Patients enrolled in the study were predominantly married (83.1%), involved in a part-time job, retired or unemployed (54.5%), had an intermediate educational level or less (54.5%), and were recruited from AUBMC hospital (52.6%). The majority of patients reported owning their house (79.8%), having enough income (82.8%), and having a crowding index ≤ 1 person/room (78.7%), reflecting a higher socioeconomic status.

**Table 1:** Demographic, socioeconomic, anthropometric, and lifestyle characteristics of CVD patients admitted for hospitalization in Lebanon^a^ (n = 367).

Demographic, socioeconomic, anthropometric, and lifestyle characteristics	Total (n = 367)
Age (years)	67 (57, 74)
**Demographic characteristics**	
Gender	
Male	260 (70.8)
Female	107 (29.2)
Marital status	
Married	305 (83.1)
Not married^b^	62 (16.9)
Site of recruitment	
AUBMC	193 (52.6)
CHN	150 (40.9)
Others^c^	24 (6.5)
**Socioeconomic characteristics**	
Educational level	
Illiterate & elementary	105 (28.6)
Intermediate	95 (25.9)
Secondary & university	167 (45.5)
Employment status	
Full time employment	148 (40.3)
Part time employment	19 (5.2)
Retired/ unemployed	200 (54.5)
Housing status	
Owned	293 (79.8)
Not owned	74 (20.2)
Crowing index	
≤ 1 person/room	289 (78.7)
>1 person/room	78 (21.3)
Perceived adequacy of income	
Enough	304 (82.8)
Not enough	63 (17.2)
Anthropometric characteristics, median (Q1, Q3)	
Weight (kg)	78 (69, 89)
Height (cm)	168 (161, 173)
BMI (kg/m^2^)	27.5 (25, 31.1)
WC (cm)	100 (91, 108)
BMI classification (kg/m^2^), n (%)	
Normal^d^	95 (25.9)
Overweight	157 (42.8)
Obese	115 (31.3)
WC classification (cm), n (%)	
Normal	37 (17.2)
Elevated	178 (82.8)
**Lifestyle characteristics**	
Cigarette smoking status	
Current smoker	119 (32.4)
Former smoker	109 (29.7)
Never smoked	139 (37.9)
Number of cigarette smoked/day for current smokers	
1–14	22 (18.3)
15–24	46 (38.3)
25+	52 (43.3)
Waterpipe smoking status	
Current smoker	30 (8.2)
Former smoker	16 (4.4)
Never smoked	321 (87.5)
Number of times smoking waterpipe/week for current smokers	
≤ 2 times	14 (46.7)
3–6 times	7 (23.3)
Daily	9 (30)
Physical activity levels	
Low	191 (52)
Moderate	109 (29.7)
High	67 (18.3)

^a^Categorical variable are expressed as n (%), continuous variables are expressed as median (Q1, Q3); Q1, first quartile; Q3, third quartile; CVD, Cardiovascular Diseases; AUBMC, American University of Beirut Medical Center; CHN, Centre Hospitalier du Nord; Kg, Kilograms; kg/m^2^, Kilograms square Meter; cm, centimeters; WC, Waist Circumference; BMI, Body Mass Index.

^b^Not married include never married, divorced, separated, or widowed.

^c^Others includes Ain Wazein Hospital (AWH) and Labib Medical Center (LMC).

^d^Normal weight includes 2 participants (0.5%) who were underweight.

Overall, almost 74% of the study sample was overweight or obese. More specifically, 42.8% were overweight and close to a third (31.1%) were obese. Median WC for the total sample was 100 (91, 108) cm, with 82.8% having an elevated WC according to the sex-specific IDF cut-offs. Most of the study sample were current (32.4%) or former (29.7%) cigarette smokers. Among those who were current cigarette smokers, more than forty percent (43.3%) reported smoking at least 25 cigarettes per day. Approximately 8% reported being current waterpipe smokers, of which 30% were daily smokers. Additionally, the results reveal that over half of the study participants (52%) had a low level of PA, 29.7% had a moderate level, and only 18.3% had a high level of PA.

#### 2 Psychosocial characteristics of the study sample

The psychometric properties of the Three-Item Loneliness Scale are shown in Table 2 for the total study sample (*n* = 367). Based on the WHO-5 items questionnaire to detect depressive symptoms, the results showed that less than a third of subjects reported being happy (25.1%), calm and peaceful (32.1%) most or all of the time. In the study sample, almost one in four subjects (25.1%) was at a high risk of mental health problems. Overall, more than half of the study participants reported hardly ever or never lacking companionship (61%) or feeling lonely (62.1%), and 42.8% reported having people they can talk to. The score of the three-item loneliness scale revealed that 34.3% of the patients were classified as “lonely” while 65.7% were classified as “not lonely”.

**Table 2:** Assessment of loneliness and mental health status amongst CVD inpatients based on the three-items loneliness scale and the WHO five items questionnaire, respectively^a^ (n = 283).

Psychosocial characteristics	Total (n = 283)
**Mental health status**	
Been a happy person	
None of the time	18 (6.4)
A little of the time	43 (15.2)
Some of the time	49 (17.3)
Good bit of the time	102 (36)
Most of the time	63 (22.3)
All of the time	8 (2.8)
Felt calm and peaceful	
None of the time	18 (6.4)
A little of the time	41 (14.5)
Some of the time	50 (17.7)
Good bit of the time	83 (29.3)
Most of the time	83 (29.3)
All of the time	8 (2.8)
Been very nervous	
None of the time	22 (7.8)
A little of the time	70 (24.7)
Some of the time	85 (30)
Good bit of the time	39 (13.8)
Most of the time	45 (15.9)
All of the time	22 (7.8)
Felt downhearted and blue	
None of the time	75 (26.5)
A little of the time	109 (38.5)
Some of the time	31 (11)
Good bit of the time	30 (10.6)
Most of the time	26 (9.2)
All of the time	12 (4.2)
Felt so down that nothing cheers you up	
None of the time	139 (49.1)
A little of the time	63 (22.3)
Some of the time	20 (7.1)
Good bit of the time	24 (8.5)
Most of the time	28 (9.9)
All of the time	9 (3.2)
**Mental health risk**	
High risk	71 (25.1)
Low risk	212 (74.9)
**Loneliness status**	
Lack companionship	
Often	19 (5.2)
Some of the time	124 (33.8)
Hardly ever or never	224 (61)
Feeling lonely	
Often	17 (4.6)
Some of the time	122 (33.2)
Hardly ever or never	228 (62.1)
Feel there are people you can talk to	
Often	157 (42.8)
Some of the time	105 (28.6)
Hardly ever or never	105 (28.6)
**Loneliness level**	
Lonely	126 (34.3)
Not lonely	241 (65.7)

^a^Categorical variables are expressed as n (%): Frequency and percentage.

#### 3 Dietary adherence in the study sample

A total of 154 patients completed the FFQ, from which 5 patients were excluded due to missing dietary intake data, and 20 patients were excluded given that their reported energy intake exceeded 6000 Kcal per day [48,49]. The total number of patients included in the dietary analysis was therefore 129 patients. Energy, nutrients, and food group intakes of the study sample are listed in Table 3. Median energy intake was estimated at 3342 Kcal. Overall, participants consumed 43.5% of their energy intake (EI) from total fats, with a higher percentage of monounsaturated (18.8%) and saturated fats (10.9%), compared to polyunsaturated fats (8%). The percentage of energy intake from carbohydrates was estimated at 42.2%, while that of protein was estimated at 13.5%.

**Table 3:** Macronutrients, sodium, and food groups intake amongst CVD patients admitted for hospitalization at AUBMC Hospital^a^ (n = 129).

	g/day	As %EI
**Macronutrients and sodium**	**Median (Q1, Q3)**	**Median (Q1, Q3)**
Energy (Kcal/d)	3342 (2713, 3983)	-
Proteins (g/d)	110.6 (90.6, 137.4)	13.5 (12.2, 15.1)
Carbohydrate (g/d)	337.5 (261.8, 427.2)	42.2 (36.7, 46.6)
Total fats (g/d)	162.2 (133.0, 190.5)	43.5 (38.7, 48.4)
Saturated fat (g/d)	40 (29.0, 53.2)	10.96 (9.0, 12.9)
Polyunsaturated fat (g/d)	32 (23.6, 41.5)	8 (6.2, 11.2)
Monounsaturated fat (g/d)	70.3 (56.4, 86.8)	18.8 (15.9, 21.5)
Cholesterol (mg/d)	274.6 (193.0, 388.0)	-
Sodium (mg/d)	2765 (2107.0, 3382.0)	-
Total dietary fiber (g/d)	34.1 (25.5, 44.2)	-
Total sugar (g/d)	103.7 (75.0, 134.0)	-
**Food groups**		
Fruits (cups/d)	1.5 (1.0, 2.0)	
Vegetables (cups/d)	1.3 (1.0, 2.6)	
Dark green vegetables (cups/w)	0.11 (0.11, 0.23)	
Red vegetables (cups/w)	3.1 (2.4, 7.7)	
Starchy vegetables (cups/w)	0.53 (0.2, 1.6)	
Other vegetables^b^ (cups/w)	4.5 (4.1, 10.4)	
Grains (oz eq/d)	13.1 (9.3, 18.2)	
Whole grains	1.3 (0.4, 5.8)	
Other grains^c^	8.33 (5.7, 14.5)	
Protein’s food (oz eq/d)	8 (6.0, 11.4)	
Lean meat, poultry, eggs (oz eq/w)	30.3 (24.1, 39.6)	
Fish (oz eq/w)	2.3 (1.0, 5.0)	
Nuts, seeds, legumes (oz eq/w)	22.1 (11.7, 37.0)	
Fat-free or low-fat dairy (cups/d)	0.44 (0.2, 1.1)	
**Adherence to the AHA recommendations**	**n (%)**	
Adherent (≥ median)	74 (57.4)	
Non-adherent (< median)	55 (42.6)	

^a^Continuous variables are expressed as median (Q1, Q3); EI, Energy Intake; CVD, Cardiovascular Diseases; AUBMC, American University of Beirut; Kcal, Kilocalories; g, gram; mg, milligrams; d, day; w, week; Oz eq, Ounce equivalent.

^b^Other vegetables include cauliflower, green salad, canned vegetables, vegetable juices, zucchini, and eggplant.

^c^Other grains include biscuits, white breads, kaak, kishek, manaeesh, pasta, pizza, white rice and toast.

On average, participants consumed 1.5 cups of fruits and 1.3 cups of vegetables per day. For the subtypes of vegetables, the lowest consumption was observed for dark green vegetables (0.11 cups/week). Amongst the grain food groups, the overall consumption of whole grains was almost six-fold lower than that of “other” grains. For the protein food groups, the consumption of lean meat, poultry, and eggs was the highest (30.3 oz/week) while the intake of fish was the lowest (2.3 oz/week). A weekly intake of 22.1 oz was observed for nuts, seeds, and legumes. On average, participants consumed less than half a cup of fat-free or low-fat milk per day. Around 43% of the study sample was found to have low dietary adherence as per the classification of the American Health Association dietary recommendation.

#### 4 Drug adherence in the study sample

Drug adherence levels of the study sample are shown in Table 4 (n = 271). Of the total sample, only 31% were classified as having high adherence to their prescribed medications.

**Table 4:** Assessment of drug adherence amongst CVD patients based on the 8-item Morisky medication adherence scale^a^ (n = 271).

Drug adherence (= Yes)	Total (n = 271)
Forget to take pill in the past two weeks	90 (33.2)
Did not take the pill in the past two weeks for other reasons	46 (17.0)
Stopped medication on your own	16 (5.9)
Forget your medicine when you leave home or travel	106 (39.1)
Took all medicines yesterday	224 (82.7)
Stop taking medication if the symptoms are under control	30 (11.1)
Feel hassled about sticking to the treatment plan	33 (12.2)
**Have difficulties remembering to take all your medicine**	
Never/rarely	174 (64.2)
Once in a while	44 (16.2)
Sometimes	48 (17.7)
Usually	5 (1.8)
All the time	0 (0)
**Drug adherence levels**	
Low adherence	74 (27.3)
Moderate adherence	113 (41.7)
High adherence	84 (31.0)

^a^Categorical variables are expressed as n (%): Frequency and percentage. CVD, Cardiovascular Diseases; O/O, Overweight/Obese; AHA, American Heart Association.

### B. Sociodemographic, anthropometric, psychosocial, and lifestyle characteristics in relation to diet adherence, drug adherence, and physical activity levels

As shown in Table 5, a significantly higher proportion of males were adhering to drug prescription and performing high PA levels as compared to females (75.0% vs. 29.5% and 80.7% vs. 19.3%, respectively). A perceived “enough income” status was significantly higher among patients who perform a low level of physical activity and those with high adherence to drug prescriptions. Compared to employed patients, unemployed patients had a significantly higher levels of low physical activity. Surprisingly, overweight or obese patients had significantly higher adherence to dietary recommendations as compared to patients with a normal weight. As compared to non-smokers, a significantly higher proportion of patients who were current or former smokers were not adherent to their drug prescriptions. Mental health risk was significantly associated with dietary and drug adherence, and the least lonely patients had high to moderate PA levels.

**Table 5:** Diet adherence, drug adherence and physical activity level among CVD patients admitted for hospitalization in Lebanon, across various sociodemographic, anthropometric, lifestyle and psychosocial characteristics^a^.

	Adherence to AHA recommendation (n = 129)				Adherence to drug prescriptions (n = 271)				Physical activity levels (n = 367)			
	Total	Non-adherent	Adherent	p-value	Total	Non-adherent	Adherent	p-value	Total	Moderate/ high	Low	p-value
**Gender**												
Male	128 (99.2)	55 (100)	73(98.6)	0.387	163(66.3)	122(70.5)	41 (56.2)	**0.03**	260(70.8)	142(80.7)	118(61.8)	**<0.001**
Female	1 (0.8)	0 (0)	1 (1.4)		83(33.7)	51(29.5)	32 (43.8)		107(29.2)	34(19.3)	73(38.2)	
**Age**												
< 60	42 (32.6)	18 (32.7)	24 (32.4)	0.972	79 (29.2)	54 (28.9)	25 (29.8)	0.882	112 (30.5)	60 (34.1)	52 (27.2)	0.154
≥ 60	87 (67.4)	37 (67.3)	50 (67.6)		192 (70.8)	133 (71.1)	59 (70.2)		255 (69.5)	116 (65.9)	139 (72.8)	
**Marital status**												
Married	111(86)	46(83.6)	65(87.8)	0.496	202(82.1)	142 (82.1)	60(82.2)	0.983	305(83.1)	150(85.2)	155(81.2)	0.298
Others	18(14)	9(16.4)	9(12.2)		44(17.9)	31(17.9)	13(17.8)		62(16.9)	26(14.8)	36(18.8)	
**Perceived income status**												
Enough	67(55.4)	30(58.8)	37(52.9)	0.514	93(39.9)	74(44.3)	19(28.8)	**0.029**	146(42)	92(55.4)	54(29.7)	**<0.001**
Not enough	54(44.6)	21(41.2)	33(47.1)		140(60.1)	93(55.7)	47(71.2)		202(58)	74(44.6)	128(70.3)	
**Educational level**												
Secondary or more	96(74.4)	42(76.4)	54(73)	0.662	104(42.3)	79(45.7)	25(34.2)	0.098	169(46)	90(51.1)	79(41.4)	0.061
Intermediate or less	33(25.6)	13(23.6)	20(27)		142(57.7)	94(54.3)	48(65.8)		198(54)	86(48.9)	112(58.6)	
**Employment status**												
Employed	67 (51.9)	30 (54.5)	37 (50)	0.609	104 (38.4)	78 (41.7)	26 (31)	0.092	148 (40.3)	92 (52.3)	56 (29.3)	**<0.001**
Unemployed	62 (48.1)	25 (45.5)	37 (50)		167 (61.1)	109 (58.3)	58 (69)		219 (59.7)	84 (47.7)	135 (70.7)	
**Housing status**												
Owned	103(79.8)	44(80)	59(79.7)	0.97	198(80.5)	132(76.3)	66(90.4)	0.1	293(79.8)	145(82.4)	148(77.5)	0.243
Not owned	26(20.2)	11(20)	15(20.3)		48(19.5)	41(23.7)	7(9.6)		74(20.2)	31(17.6)	43(22.5)	
**Crowding index**												
≤ 1 person per room	115(89.1)	47(85.5)	68(91.9)	0.245	195(79.3)	142(82.1)	53(72.6)	0.094	289(78.7)	137(77.8)	152(79.6)	0.684
> 1 person per room	14(10.9)	8(14.5)	6(8.1)		51(20.7)	31(17.9)	20(27.4)		78(21.3)	39(22.2)	39(20.4)	
**BMI classification**												
Normal weight	25(19.5)	16(29.1)	9(12.3)	**0.018**	62(25.4)	40(23.3)	22(30.6)	0.232	95(26.2)	49(28)	46(24.6)	0.462
Overweight or obese	103(80.5)	39(70.9)	64(87.7)		182(74.6)	132(76.7)	50(69.4)		267(73.8)	126(72)	141(75.4)	
**WC classification**												
Normal	5(14.3)	3(20)	2(10)	0.403	27(16.5)	17(17.9)	10(14.5)	0.562	37(17.2)	22(21.2)	15(13.5)	0.138
Elevated	30(85.7)	12(80)	18(90)		137(83.5)	78(82.1)	59(85.5)		178(82.8)	82(78.8)	96(86.5)	
**Smoking status**												
Never	26(20.2)	9(16.4)	17(23)	0.355	92(33.9)	56(29.9)	36 (42.9)	**0.038**	112(30.9)	50(28.4)	62(33.3)	0.311
Current/Past	103(79.8)	46(83.6)	57(77)		179(66.1)	131(70.1)	48 (57.1)		250(69.1)	126(71.6)	124(66.7)	
**Mental Health risk**												
Low risk	38 (46.9)	9 (29)	29 (58)	**0.011**	203 (74.9)	127 (67.9)	76 (90.5)	**<0.001**	212 (74.9)	105 (77.2)	107 (72.8)	0.392
High risk	43 (53.1)	22 (71)	21 (42)		68 (25.1)	60 (32.1)	8 (9.5)		71 (25.1)	31 (22.8)	40 (27.2)	
**Loneliness level**												
Not lonely	109 (84.5)	44 (80)	65 (87.8)	0.224	159 (59.1)	113 (60.4)	46 (56.1)	0.506	233 (65.1)	128 (73.6)	105 (57.1)	**0.001**
Lonely	20 (15.5)	11 (20)	9 (12.2)		110 (40.9)	74 (39.6)	36 (43.9)		125 (34.9)	46 (26.4)	79 (42.9)	

Significance is derived from chi-square test (χ^2^) or fisher exact test. Bold values indicate significant at *p* < 0.05.

^a^Categorical variables are expressed as n (%): Frequency and percentage. CVD, Cardiovascular Diseases; O/O, Overweight/Obese; AHA, American Heart Association.

Table 6 shows the predictors of adherence to dietary, drugs, and physical activity recommendations based on multivariable logistic regression models. In the multivariable model, patients who had a high mental health risk had almost five times lower odds of being adherent to dietary recommendations when compared to patients with low mental health risk (AOR = 0.2; 95% CI: 0.06, 0.6). Similarly, a high mental health risk was significantly associated with lower adherence to drug prescriptions (AOR = 0.27; 95% CI 0.12, 0.6). Remarkably, being overweight or obese remained a significant predictor of dietary adherence after adjusting for potential confounders (AOR = 5.9, 95% CI: 1.4, 25.9). However, no significant associations were observed between each of smoking status, perceived income status, and gender with drug adherence after controlling for confounders. As for physical activity, loneliness remained significantly associated with lower odds of engaging in high PA levels (AOR = 0.5, 95%CI: 0.31,0.8), while male gender remained significantly associated with higher odds of engaging in PA levels (AOR = 2.5, 95%CI: 1.4, 4.3). Employment and perceived income status were no longer significant predictors of PA.

**Table 6:** Predictors of adherence to dietary, drug, and physical activity recommendations among CVD patients as assessed by multiple logistic regression.

Variables	Adjusted OR (AOR)	95% CI	p-value
**High adherence to dietary recommendations** ^a^			
Mental Health status			0.010
Low risk	1 [ref]		
High risk	0.2	0.07, 0.7	
BMI status			0.018
Normal	1 [ref]		
O/O	5.9	1.4, 25.9	
**High adherence to drug prescriptions** ^b^			
Mental Health status			0.008
Low risk	1 [ref]		
High risk	0.274	0.12, 0.6	
**High/moderate adherence to PA level** ^c^			
Gender			<0.001
Female	1 [ref]		
Male	2.5	1.4, 4.3	
Loneliness risk			0.003
Not lonely	1 [ref]		
Lonely	0.5	0.31, 0.8	

AHA, American Heart Association; PA, Physical Activity; AOR, adjusted odds ratio; CI, Confidence Intervals; Ref, reference.

^a^This model adjusted for age, marital status, smoking status, BMI, income status, housing status, mental health status, loneliness scale, and physical activity levels.

^b^This model included marital status, age, gender, smoking status, BMI, income status, housing status, mental health status, loneliness scale, and physical activity level.

^c^This model included age, marital status, gender, smoking status, BMI, income status, housing status, loneliness scale.

Only significant results are shown in the table.

## Discussion

This study has provided insight on various sociodemographic, lifestyle and psychosocial characteristics of Lebanese CVD patients, and assessed their adherence to dietary guidelines, drug prescriptions, and PA recommendations. It showed that dietary and drug adherence rates were low in this patient population (57% and 31%, respectively) and that close to half did not follow the PA recommendations. Importantly the study identified psychosocial characteristics (i.e., mental health status and degree of loneliness) as independent predictors of the patient’s adherence to dietary, drug, and PA recommendations. This study has also underlined gender and anthropometric status as important modulators of adherence to dietary and PA recommendations.

Several previous studies have documented the clinical characteristics of patients with CVD, but very few have extended their investigation to broader socioeconomic and psychosocial factors. In 2015, the AHA published a scientific statement calling for greater attention to the social and behavioral determinants of CVD [6]. This study, therefore, responds to this call by contributing to a broader characterization of CVD inpatients. The study findings showed that the prevalence rates of low educational attainment, unemployment, smoking, as well as physical inactivity, and overweight/ obesity, were all higher in our sample of CVD patients compared to those reported for the general Lebanese population [7]. Other studies conducted in the USA and on Asian Indians have also reported similar observations [11,50].

In the present study, the diet of the study participants was assessed using a previously validated FFQ, and median caloric intake (3342Kcal) was similar to that reported by a recent study conducted among urban adults in Lebanon [51]. The percentage of energy intake from total fats (43.5%) and saturated fats (11%) were considerably high, exceeding the dietary guidelines for a heart-healthy diet [38]. This is not the first study that reports a high intake of fat and saturated fat amongst Lebanese adults. In fact, total fat intake was reported to range between 37.15% and 41.22% [35,51,52] amongst Lebanese adults and saturated fat intake was estimated to range between 12% and 17% of total energy [51]. It is however noteworthy that in this sample of CVD patients, total fat intake was higher than that reported for the general population [53]. Additionally, adherence to fruits (19.4%), vegetables (3.9%), whole grains (32.6%), fish (14.7%), low-fat or fat-free dairy (3.1%), and fibers (22.5%) intake recommendations were low, far below the international recommendations [38,54]. In Lebanon, the consistent rise in daily energy and fat intakes coupled with a decrease in fruit and vegetable intake was attributed to the nutrition transition and the increased adoption of the western diet and lifestyle [7,55].

In this study, almost 57% of cardiac patients were found to be adherent to diet. Although there are no studies that previously compared dietary intake to the AHA dietary recommendations, other studies that assessed diet adherence amongst cardiac patients in Pakistan (53%) and Kuwait (63%) reported similar estimates to our study [56,57]. Although adherence to diet is complex and multifaceted, several studies suggested the incorporation of dietary recommendations into cardiac rehabilitation programs, yielding promising results in improving CVD risk factors and outcomes [58,59].

In our study, 31% of the study sample had high adherence to their drug prescriptions while the majority (69%) had low or moderate adherence. This estimate (69%) is higher than that reported amongst CVD patients from several developed and developing countries such as Sudan (51%), Saudi Arabia (47%), and Pakistan (23.5%) [41,60–65]. A meta-analysis including 76 studies from developing countries, showed that the pooled percentage of drug non-adherence amongst CVD patients was 42.5% (95% CI: 50–64%), a value that is lower than the estimate obtained in our study [18]. The disparities found between countries could be explained by differences in cultural factors, types of CVD studied, sampling design, patients’ socioeconomic characteristics, and the instrument used to measure drug adherence. Unlike our study, several studies have used self-reported methods to assess adherence levels, which could potentially overestimate drug adherence [66,67]. The level of drug non-adherence amongst cardiac patients in this study was considerably higher than that reported by a prior study conducted amongst hypertensive patients in Lebanon (22.4%) [68]. Although both studies have used the same MMAS-8 to assess drug adherence levels, the aforementioned study assessed the level of drug adherence amongst outpatients, thus, representing the least severe cases with a less complex drug regimen when compared to our study participants.

In the current study, the rates of physical inactivity (52%) were lower than estimates reported amongst CVD patients from other countries in the regions such as Kuwait (62.6%) and Saudi Arabia (67.6%) but higher than Syria (39.6%) and Sweden (32%) [69,70]. The discrepancies in the results could be due to differences in the sampling design as well as the instruments and scoring methods used to assess PA levels. Unlike our study, the majority of the above-mentioned studies were conducted among patients with heart failure, thus, representing severe cardiac cases with a lower ability to engage in high-intensity PA [71]. The proportion of patients with the high level of PA was estimated at 18.3%, in our study sample which is comparable to the figures previously reported by Sibai et al. and Isma’eel et al., where almost 18% and 21% of cardiac patients were engaged in high PA levels, respectively [72,73].

A higher sense of well-being was found to be a significant predictor of higher diet adherence in our study. These results are consistent with a recent cross-sectional study showing a negative correlation between depression and diet adherence amongst CVD patients [46]. More specifically, the same study reported that long-term adherence to dietary recommendations may be difficult for patients with depression, especially among men [46]. Therefore, it is important to consider psychosocial factors and their potential impact on CVD risk factors to delay disease progression and improve treatment response and patient’s quality of life [46]. Surprisingly, the results of this study showed that higher BMI was associated with higher dietary adherence. These observations do not support the findings of previous studies, which have suggested that low adherence to dietary recommendations is associated with overweight and obesity [74–76]. In the present study, the possibility of reverse causality may have affected our results. In other words, overweight or obese patients could have intentionally improved their dietary practices and hence their adherence to a healthier diet. Another explanation could be attributed to the phenomenon of obesity-related dietary underreporting especially for energy-dense, high-fat or high-sugar foods [77–80]. In fact, several studies have previously shown that underreporting is directly and significantly associated with higher BMI, particularly among patients suffering from chronic illness such as diabetes and hypertension [78–81].

Similar to the findings reported by the Heart and Soul Study, the multivariable analyses model showed that a high mental health risk was a significant predictor of drug non-adherence in CVD inpatients. Although the precise mechanism by which poor mental health affects drug compliance is still unclear and complex, studies suggested possible explanations for this relationship [82], For instance, patients with a high mental health risk may be more sensitive to medication adverse effects or feel hopeless as to the treatment benefits [83]. They may also have lower self-care practices, are less likely to have the energy to focus on treatment recommendations, and may even engage in intentional self-harm [83]. Consequently, these patients are more likely to discontinue medication use [83]. In our study, medication non-adherence was not significantly associated with socioeconomic or lifestyle characteristics. These findings are consistent with studies conducted in China and Lebanon [62,68] but not with studies undertaken in France [84].

The predictors of high PA included gender and lower loneliness levels. The least lonely patients had in fact higher PA levels, similar to the findings reported by Darden et al., where loneliness was significantly and inversely associated with the frequency and duration of PA [85]. As expected, male gender was also a strong predictor of high PA level, which is consistent with findings reported from several studies conducted in neighborhood countries such as Egypt, KSA, and Qatar, whereas studies undertaken in Sweden did not report such observations [69,86]. In some countries of the EMR, the low levels of PA amongst females reflect the cultural and religious factors discouraging women from engaging in physical activity [87].

Overall, our study has shown that CVD patients in Lebanon have poor dietary and drug adherence and that less than half engage in PA as per the recommendations. These findings are in agreement with reports from the WHO, according to which almost 50% of patients with chronic diseases are not adherent to their treatment regimen which includes diet, drug, and physical activity [88]. In our study, this low level of adherence was observed despite the fact that a high proportion of patients were from a higher socioeconomic status (based on crowding index and house ownership). Our results may therefore be an underestimate of the true level of non-adherence in the country. Although it may have been expected to find an association between socioeconomic status and adherence to dietary or drug regimens amongst CVD patients, this association was not observed in our study [56,89–94]. Our results have in fact shown that, even after adjustment for socioeconomic correlates, psychosocial characteristics such as loneliness and mental health remained independent predictors of adherence in this sample of CVD patients. Therefore, this study’s findings indicate that psychosocial characteristics, especially loneliness and low mental well-being, could be potential root causes of low drug, diet, and physical activity adherence that should be taken into account for the treatment of patients with CVD.

The results of this study should be considered in light of the following limitations. First, this is a cross-sectional study that allowed us to assess the association rather than any causal relationship. Second, the recruitment of subjects was performed in the hospital setting and thus the results cannot be directly generalized or extrapolated to the general population of CVD patients in Lebanon. Third, considering that AUBMC is the largest hospital in Lebanon and that the size of the data collection teams differed between hospital sites, almost half of the surveyed patients were recruited from AUBMC. It is however important to clarify that the patient body at AUBMC is not homogeneous as this major hospital attracts patients from the various regions in Lebanon [17], and this was reflected in the results of our study: 56% of the recruited patients at AUBMC were from the capital Beirut, while the rest of the patients were from other governorates (data included in S1 File). The fact that a high proportion of subjects were from a higher socioeconomic status could have resulted in an underestimate of non-adherence. Fourth, information on the duration of the disease was not collected from the recruited participants and thus, we were not able to differentiate between adherence levels amongst those who had longer vs shorter illness duration. Fifth, this study relied on an interview-based approach for the collection of data, which may be limited by the possibility of social desirability bias. The FFQ may also be limited by recall bias and difficulties in portion size estimation. Despite these limitations, the FFQ was validated in the Lebanese population and is considered amongst the most suitable dietary assessment tools to assess the subject’s habitual diet over a longer period of time [34]. The FFQ was also administered by experienced dietitians who have been trained to reduce judgmental verbal and non-verbal communication to obtain information about the foods usually consumed by the individual, which should have enhanced the quality of the data collected.

## Conclusion

This study is amongst the few in the EMR region to investigate the determinant of diet adherence, drug adherence, and physical activity amongst hospitalized cardiac patients. It showed that adherence to dietary, drug and PA recommendations were low in the study sample and that psychosocial characteristics that include loneliness, and poor mental well-being were predictors of medication, diet, and PA non-adherence. Our findings should thus direct attention toward psychosocial characteristics that should be taken into account for the treatment of patients with CVD. The results of this study may thus provide evidence of the importance of identifying nonclinical patient characteristics, and how they potentially constellate, to provide actionable knowledge that may be used to develop effective approaches to enhance the quality of diets in CVD patients. The study findings may lay the groundwork for further studies that have the potential to assist decision-makers in developing primary and secondary prevention strategies related to CVD.

## Supporting information

S1 File (XLSX)
